# Longitudinal investigation of neuroinflammation and metabolite profiles in the APP_swe_×PS1_Δe9_ transgenic mouse model of Alzheimer's disease

**DOI:** 10.1111/jnc.14251

**Published:** 2017-12-15

**Authors:** Aisling Chaney, Martin Bauer, Daniela Bochicchio, Alison Smigova, Michael Kassiou, Karen E. Davies, Steve R. Williams, Herve Boutin

**Affiliations:** ^1^ Centre for Imaging Science Faculty of Biology, Medicine and Health Manchester Academic Health Sciences Centre University of Manchester Manchester UK; ^2^ Wolfson Molecular Imaging Centre Faculty of Biology, Medicine and Health and Manchester Academic Health Sciences Centre University of Manchester Manchester UK; ^3^ Department of Clinical Pharmacology Medical University Vienna Vienna Austria; ^4^ School of Chemistry University of Sydney NSW Australia; ^5^ Present address: 3165 Porter Drive Stanford University Palo Alto CA 94304

**Keywords:** Alzheimer's disease, animal model, magnetic resonance spectroscopy, neuroinflammation, positron emission tomography

## Abstract

There is increasing evidence linking neuroinflammation to many neurological disorders including Alzheimer's disease (AD); however, its exact contribution to disease manifestation and/or progression is poorly understood. Therefore, there is a need to investigate neuroinflammation in both health and disease. Here, we investigate cognitive decline, neuroinflammatory and other pathophysiological changes in the APP_swe_×PS1_Δe9_ transgenic mouse model of AD. Transgenic (TG) mice were compared to C57BL/6 wild type (WT) mice at 6, 12 and 18 months of age. Neuroinflammation was investigated by [^18^F]DPA‐714 positron emission tomography and *myo*‐inositol levels using ^1^H magnetic resonance spectroscopy (MRS) *in vivo*. Neuronal and cellular dysfunction was investigated by looking at N‐acetylaspartate (NAA), choline‐containing compounds, taurine and glutamate also using MRS. Cognitive decline was first observed at 12 m of age in the TG mice as assessed by working memory tests . A significant increase in [^18^F]DPA‐714 uptake was seen in the hippocampus and cortex of 18 m‐old TG mice when compared to age‐matched WT mice and 6 m‐old TG mice. No overall effect of gene was seen on metabolite levels; however, a significant reduction in NAA was observed in 18 m‐old TG mice when compared to WT. In addition, age resulted in a decrease in glutamate and an increase in choline levels. Therefore, we can conclude that increased neuroinflammation and cognitive decline are observed in TG animals, whereas NAA alterations occurring with age are exacerbated in the TG mice. These results support the role of neuroinflammation and metabolite alteration in AD and in ageing.

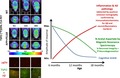

**Abbreviations used**
AD: Alzheimer's disease
Aβ: β‐amyloid plaques
CD11b: integrin αM chain of the Macrophage‐1 antigen
CSF: cerebrospinal fluid
Cr: creatine & phosphocreatine
DI: discrimination index
Tf: time spent exploring the familiar object/scent
GFAP: glial fibrillary acidic protein
Glu: glutamate
MRS: magnetic resonance spectroscopy
MAP2: microtubule‐associated protein‐2
MCI: mild cognitive impairment
MR: magnetic resonance
mI: myo‐inositol
NAA: N‐acetylaspartate
NFTs: neurofibrillary tangles
NUVcb: normalized (to the cerebellum) uptake value
NOR: novel object recognition
NSR: novel smell recognition
PET: positron emission tomography
SUV: standard uptake value
SVA2: synaptic vesicle glycoprotein 2A
Taur: taurine
Tn: time spent exploring the novel object/scent
tCho: total choline
T: total time exploring objects/scents
TG: transgenic
TSPO: translocator receptor 18 kDa
WT: wild type

Alzheimer's disease (AD) is the most common form of dementia which has become a huge socio‐economic burden because of an increased ageing population. Despite considerable research, the mechanisms leading to its manifestation remain unclear. AD is defined pathologically by amyloid plaques (Aβ), neurofibrillary tangles and neuronal loss (Braak and Braak 1991); however, the aetiology remains elusive. Hence, a need exists to find biomarkers that can help understand disease progression and mechanisms, aid diagnosis and track the efficacy of therapeutic interventions.

AD is a multifactorial disease associated with complex neuronal and neuroinflammatory alterations (Halliday *et al*. 2000; Rubio‐Perez and Morillas‐Ruiz 2012; Perry and Holmes 2014; Heneka *et al*. 2015; Mhatre *et al*. 2015). Activated microglia have been shown to surround amyloid plaques (Haga *et al*. 1989) *ex vivo* and increased inflammatory cytokine expression is observed post‐mortem in brain and *in vivo* in the serum and CSF of AD and patients with mild cognitive impairment (MCI) (Forlenza *et al*. 2009; Swardfager *et al*. 2010; Rubio‐Perez and Morillas‐Ruiz 2012; Varnum and Ikezu 2012; Westin *et al*. 2012). In addition, treatments which experimentally reduce pro‐inflammatory cytokines have been shown to improve memory performance (Lee *et al*. 2013; Song *et al*. 2013), suggesting increased inflammation could contribute to memory impairments seen in AD.

The ability to accurately assess neuroinflammation *in vivo* is important to our understanding of the impact it has in AD. The discovery that translocator receptor 18 kDa (TSPO) expression is up‐regulated on activated microglial cells (Papadopoulos *et al*. 2006) has enabled the generation of TSPO radio‐ligands to image neuroinflammation *in vivo* using positron emission tomography (PET). TSPO expression in the brain is mainly restricted to endothelial cells and activated microglia, and is low in healthy brain tissue where inflammation is absent (Papadopoulos *et al*. 2006; Scarf and Kassiou 2011); therefore TSPO PET permits the identification and tracking of region‐specific neuroinflammation throughout disease progression. It is important to note that TSPO is also expressed in endothelial cells and hence in blood vessels (Turkheimer *et al*. 2007).

PK11195, a TSPO ligand, has been successfully labelled with ^11^C and has shown good correlation to microglial activation *ex vivo* (Venneti *et al*. 2009); however, contradictory reports exist regarding the detection of neuroinflammation in AD using [^11^C]PK11195 imaging. Some studies have demonstrated increased [^11^C]PK11195 binding in AD and MCI patients (Edison *et al*. 2008; Yokokura *et al*. 2011), whereas others have reported no differences in any regions between patients and age‐matched controls (Wiley *et al*. 2009; Schuitemaker *et al*. 2013). Short half‐life, low signal to noise ratio, high non‐specific binding and modelling issues of [^11^C]PK11195 may have contributed to these contradictory results (Boutin and Pinborg 2015; Varrone and Lammertsma 2015); hence, new generation tracers with improved affinities and kinetics such as [^18^F]DPA‐714 (James *et al*. 2008; Chauveau *et al*. 2009; Doorduin *et al*. 2009; Boutin *et al*. 2013) have been developed to assess. Recently, using [^18^F]DPA‐714, Hamelin *et al*. (2016) have demonstrated that AD patients with slower cognitive decline had higher level of neuroinflammation, hypothesizing that neuroinflammation may also exert neuroprotective functions early in the disease. However, this is the first *in vivo* study showing a positive correlation between neuroinflammation and cognitive decline, suggesting a potential beneficial role for neuroinflammation in AD. However, this needs to be further investigated as TSPO expression is only a surrogate marker of microglial activation in a broad sense and does not provide information on the functional phenotype of activated microglia. Similarly, TSPO expression has been reported to be increased with ageing (Gulyas *et al*. 2011; Kumar *et al*. 2012) but the role and meaning of this potential increase with age is yet to be fully understood.

Similarly, magnetic resonance spectroscopy (MRS), a method that allows the detection of metabolite profiles *in vivo*, can also be used to investigate neuroinflammation through measurement of myo‐inositol (mI) levels. mI has been suggested to be a glial specific marker (Brand *et al*. 1993) indicative of increased gliosis. Although increased levels of mI have been repeatedly reported in AD and MCI subjects in regions associated with degeneration in AD (Parnetti *et al*. 1997; Rose *et al*. 1999; Kantarci 2007; Shinno *et al*. 2007; Foy *et al*. 2011; Shiino *et al*. 2012; Murray *et al*. 2014), evidence suggests that mI does not necessarily correlate with increased microglial activation *ex vivo* (Murray *et al*. 2014; Pardon *et al*. 2016) and may be more directly associated with plaque load. MRS can also measure other metabolites of interest related to neuronal function including N‐acetylaspartate (NAA), glutamate (Glu), taurine (Taur), total choline (tCho) and creatine + phosphocreatine (Cr). NAA is a neuronal marker (Birken and Oldendorf 1989; Urenjak *et al*. 1993) and decreases in its levels are consistently reported in AD patients (Parnetti *et al*. 1997; Rose *et al*. 1999; Kantarci 2007; Shinno *et al*. 2007; Watanabe *et al*. 2010; Foy *et al*. 2011; Shiino *et al*. 2012; Murray *et al*. 2014), suggesting neuronal dysfunction or death. Moreover, mI and NAA levels have been shown to be correlated with Aβ burden (Klunk *et al*. 1992; Murray *et al*. 2014) and performance in some cognitive tasks (Rose *et al*. 1999; Foy *et al*. 2011), implicating both in AD pathology and disease severity.

Although, the majority of AD cases are sporadic, so far most of the animal models use transgenes present in the inheritable familial form. Nevertheless, these models give us insight into the pathological mechanisms underlying AD. Hence, here we used the human double mutation APP_swe_×PS1_Δe9_ mouse model to investigate inflammatory and neuronal integrity biomarkers as indicators of disease progression using MRS, PET, immunohistochemistry and cognitive assessment.

A longitudinal study was conducted in male TG APP_swe_×PS1_Δe9_ and WT C57BL/6 mice to investigate neuroinflammation using PET, neurochemical profile using MRS and cognitive function using several behavioural assessments. This was done with the aim of characterizing inflammatory, metabolite and cognitive differences between TG and control animals with age. We hypothesized that there would be increased neuroinflammation (demonstrated by increased DPA‐714 uptake and mI levels), neuronal dysfunction (decreased NAA levels), neurotransmitter disturbances (altered Glu) and cognitive decline with age in the TG compared to WT mice.

## Methods

### Animals

Fourteen TG male APP_swe_×PS1_Δe9_ mice (RRID: MGI:5701399) with a C57BL/6 (RRID: IMSR_JAX:000664) background and 17 WT C57BL/6 mice were acquired from the Jackson laboratory (Bar Harbor, ME, USA) at 8 weeks of age and allocated to the study. Additionally, 5 WT and 7 TG mice were bred in‐house and complemented the initial groups (see details below). As the mice are *per se* WT or TG, the WT and TG cannot be randomized. All mice were kept in the Biological Sciences Facility in the University of Manchester. Animals were housed in individually ventilated cages in groups of 2–5 in a 12 h:12 h light and dark cycle with environmental enrichment and 24 h access to food and water. In total, 22 male WT and 21 TG male mice were used in this study. Eight WT and 10 TG were measured repeatedly for MRS (two WT and five TG of these were bred in‐house); a total of 20 WT and 16 TG (five WT and seven TG of these were bred in‐house) mice were scanned non‐repeatedly with PET. This was as a result of seven TGs and five WTs dying of unknown causes or during anaesthesia, and one WT was excluded after abnormal brain morphology was noticed during MR imaging. In addition, three animals in each group were used for analysis *ex vivo* at both 6 and 12 months. At 18 months, all mice were killed and the brains removed for analysis *ex vivo*. All experiments were carried out in accordance with the Animal Scientific Procedures act 1986 and approved by the University of Manchester Local Ethical Review Committee.

### Study design

**Figure 1:**
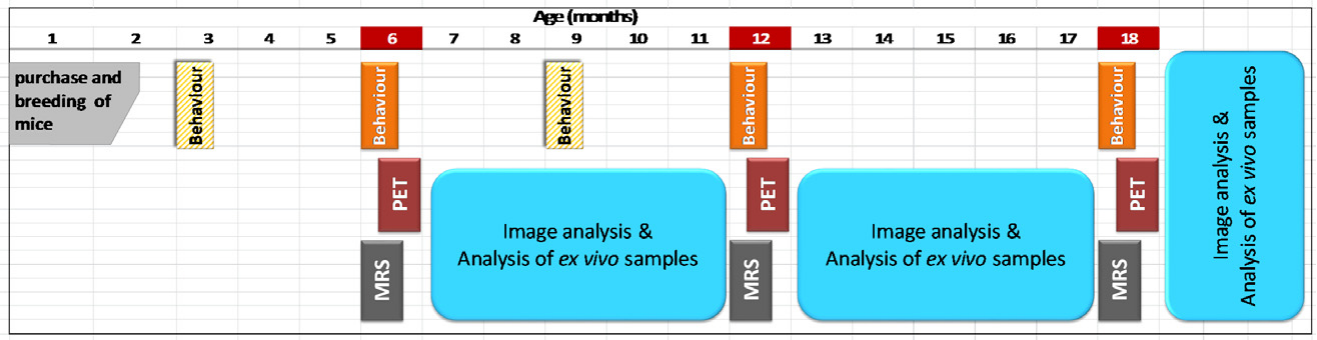
Gantt chart of the study. Behaviour tests and positron emission tomography (PET) and magnetic resonance spectroscopy (MRS) scans were performed at 6, 12 and 18 months of age. Behaviour tests were also performed at two additional time‐points to evaluate potential cognitive changes and had cognitive deficit been detected at 9 months, the imaging time‐points would have been brought forward.

The study was not pre‐registered. Mice underwent longitudinal behavioural testing and imaging (Fig. 1) at 6, 12 and 18 months of age (body weights (in grams, mean ± SD): 32.9 ± 1.71, 37.9 ± 3.45, 38.9 ± 3.69 in WT and 34.5 ± 3.19, 40.2 ± 4.28, 41.1 ± 6.17 in TG at 6 m, 12 m, 18 m of age respectively). Mice were also tested at 3 (11 WT and 15 TG) and 9 months (9 WT and 12 TG) of age to check for potential changes in behaviour and potentially guide changes in the choice of imaging time‐points; however, as there was no significant differences between 3, 6 and 9 months’ time‐points, data from the 3 and 9 months’ time‐points are not shown here and only the time‐points matching the imaging time‐points are shown. Imaging experiments began at 6 months of age because of the development of detectable amyloid pathology from this age in this model (Jankowsky *et al*. 2004). Immunohistochemistry was carried out on separate animals at 6 and 12 months (see ‘animals’ above). Animals followed longitudinally were culled and processed for *ex vivo* analysis at 18 months of age. A week gap was given between imaging and behavioural experiments to allow animals to recover from the potential stress of imaging/anaesthesia and limit interference between experiments. All experiments were carried out between 9 am and 5 pm, with all behaviour carried out between 9 am and 2 pm for each time‐point. For all behavioural tests animals were placed in the testing room 30 min prior to experimentation to allow for habituation to the environment.

### Behaviour

#### Novel object and smell recognition tests

A plastic circular arena (30 cm diameter, 21 cm height) was used for behavioural testing. Animals were subjected to 2 days of habituation and 1 day of novel object recognition (NOR) testing. Habituation involved single mice exploring an empty arena for 5 min and was carried out in a random order within the same time‐frame each day. NOR utilizes the natural behaviour of rodents to explore novelty to test non‐associative working memory (Ennaceur and Delacour 1988). This was assessed by investigating the ability to discriminate between novel and familiar objects and was carried out in both TG and WT mice at 6, 12 and 18 months of age. Small plastic objects of varying shapes and colours (e.g. Lego blocks) were used as NOR objects. Test day consisted of two phases. In phase 1, a mouse was placed in the arena with two identical unfamiliar objects for 10 min. After this time, the mouse was returned to its home cage and a 1 h delay was implemented. In phase 2, one familiar object was replaced by a novel object and the mouse was given 4 min to investigate the objects.

Novel smell recognition (NSR) utilizes the natural exploration of novelty to test working memory in rodents although through olfaction. The experimental design was the same as the NOR; however, a 3‐min delay was implemented and identical plastic scent balls with sniffing holes were stuffed with cotton wool and filled with 0.5 mL of a certain scent (e.g. vanilla) were used.

For both the NOR and NSR, behaviour was recorded and analysed retrospectively. In addition, in between trials, all objects and areas were cleaned with 70% ethanol to remove any scent of the previous mouse which may alter results.

Time spent investigating left and right identical objects/smells in phase 1 were analysed to assess side bias. Mice were to be excluded if significant side bias (> 60% of time spent investigating one of two identical objects) was observed in phase 1. Time spent exploring the novel and familiar objects in phase 2 was used to generate a discrimination index (DI), defined as the difference between time spent exploring the novel (T_n_) and the familiar (T_f_) object/scent divided by the total time (T) (DI = (T_n_‐T_f_)/T). This resulted in values ranging from −1 to +1. A negative value indicated more time spent with the familiar, a positive value indicated more time investigating the novel and a zero value indicated no preference.

The re‐use of novel objects for each mouse was kept to a minimum to prevent potential memorization of objects/scents between time‐points and ensure the test was unique (i.e. a truly novel object or smell they were never exposed to before). The only exception to this was a small cohort of mice that underwent behavioural testing at 3 months of age to investigate whether early time‐points were needed for baseline. No significant differences were observed at this age in this small group and therefore only 6, 12 and 18 months were chosen going forward with behavioural testing.

#### Y‐maze spontaneous alteration task

The Y‐maze spontaneous alteration task utilizes the natural exploratory behaviour of rodents to assess spatial learning and short‐term memory. The Y‐maze test was carried out as described previously shown by Martins *et al*. (2017). A black Perspex maze with three arms (15 cm length × 10 cm width × 10 cm depth per arm) labelled with A‐C and with different internal visual cues was used. The mice were placed inside the maze and arm entries were recorded manually over an 8‐min time period, with entries only valid if the whole body of the mouse entered the arm. Successful spontaneous alternation was defined by consecutive entry into all three arms in any order. Analysis was carried out by calculating overlapping triplet sets relative to successful arm entries as previously described (Hiramatsu *et al*. 1997; Knight *et al*. 2014). Percentage of successful alternation was compared between WT and TG mice at all ages.

#### Behaviour to predator and non‐predator urine

A Y‐maze with either predator or non‐predator urine at the end of each arm was used to assess the general olfactory ability of 12‐month‐old WT and TG mice. The maze had no visual cues. Vented containers were injected with 1 mL of bobcat, fox or rabbit urine and were placed at the end of each arm. Mice were allowed to explore the maze for 8 min and time (in seconds) spent in each arm was quantified. Time spent in the middle of or rearing on the side of the maze was not counted. Both bobcats and foxes are predators for mice, whereas rabbits are not, hence an increased amount of time in the arm containing rabbit urine would be expected in mice with intact olfaction.

### [^18^f]dpa‐714 pet

Neuroinflammation was investigated using the TSPO tracer [^18^F]DPA‐714. [^18^F]DPA‐714 was produced as previously described (James *et al*. 2008). Animals were anesthetised, cannulated (via tail vein) and injected with [^18^F]DPA‐714 (12.3 ± 1.9 MBq). Respiratory rate and temperature were monitored throughout the experiment and body temperature was maintained at 37 ± 0.5°C (BioVet^®^ system m2 m Imaging Corp., Cleveland, OH, USA). Images were acquired on a Siemens Inveon^®^ PET‐CT scanner using a 60‐min dynamic acquisition. CT scans were performed prior to PET acquisition to obtain the attenuation correction map. The time coincidence window was set to 3.432 ns and levels of energy discrimination to 350 keV and 650 keV. List mode data from emission scans were histogrammed into 16 dynamic frames (5 × 1 min; 5 × 2 min; 3 × 5 min and 3 × 10 min) and emission sinograms were normalized, corrected for attenuation, scattering and radioactivity decay and reconstructed using an OSEM3D protocol (16 subsets and 4 iterations) into images of dimensions 128 (transaxially) ×159 (axially) with 0.776 × 0.776 × 0.796 mm voxels. The PET images segmented using the local means analysis method and the organ mean time activity curves were corrected for partial volume effect as previously described (Maroy *et al*. 2008, 2010; Boutin *et al*. 2013). The correction method combined the geometric transfer matrix method and the regions of interest (ROI)‐opt method. Dynamic PET images were analysed using Brainvisa and Anatomist software (http://brainvisa.info/web/index.html) and quantified using the magnetic resonance imaging (MRI) template (Waxholm space) created by Johnson *et al*. (2010). This MRI mouse brain template was used to create three brain ROIs (all cortical areas and whole hippocampus, subcortical regions and cerebellum) large enough to be accurately quantified based on the spatial resolution of the PET scanner (Figure S1a–c). Data are expressed as uptake values normalized to the cerebellum (NUV_cb_) as previously used in the same mouse model (Serriere *et al*. 2015).

### Magnetic resonance spectroscopy acquisition and analysis

Animals were anesthetised using isoflurane (3% induction and 1–2% maintenance) and medical oxygen at a rate of 2 L/min. Respiratory rate and temperature were monitored throughout the experiments which were conducted using a 7 Tesla magnet connected to a Bruker Avance III console (Bruker Biospin Ltd, UK). An anatomical multi‐slice FLASH MRI sequence was used to enable positioning of the hippocampal volume for MRS. Spectra were acquired using a water‐suppressed PRESS sequence (Bottomley 1987) (TR 2500 ms, TE 20 ms, 512 averages) from a 2.5 × 4.5 x 3 mm^3^ voxel that covered the hippocampus and the most dorsal part of the thalamus (Figure S1d). Prior to acquiring the spectrum the localized voxel was shimmed using ‘FASTMAP’ (Gruetter 1993) and water suppression was optimized using VAPOR (Griffey and Flamig 1990). A non‐water‐suppressed reference PRESS spectrum was also acquired (1 average).

A metabolite basis set was simulated using NMRScope with the same spectroscopic parameters used for the PRESS acquisition (jMRUI version 5) (Stefan *et al*. 2009). Metabolites included in the basis‐set were: NAA, Glu, mL, Cre, GABA, scyllo‐inositol (Scy‐I), Gln, Tau and tCho. Peaks at 0.9 and 1.3 ppm were included to model lipid/macromolecules. Another additional peak was added at 3.76 ppm to cover the detection of α‐protons of amino acids not otherwise included in the basis‐set. These additional peaks were added to help with spectral fitting as significant residual signal had previously been found at these resonances (Forster *et al*. 2013). An example of an *in vivo* spectrum with labelled metabolites is shown in Fig. 1e. Spectra were pre‐processed by applying a HLSVD (Hankel Lanczos Singular Values Decomposition) filter to suppress the residual water signal (van den Boogaart *et al*. 1994). Metabolite concentration *in vivo* was measured using the jMRUI version 5 algorithm QUEST (Ratiney *et al*. 2005). QUEST compiles the metabolite theoretical signals into a basis‐set, and then fits the signals to the spectra *in vivo*, allowing detection and measurement. QUEST was run without background handling. Results were referenced to Cr. Referenced data was used to compare metabolite levels in WT compared to TG mice.

### Tissue collection and immunohistochemistry

Immunohistochemistry was carried out on TG and WT animals (*n* = 3–5) to visualize integrin αM chain of the Macrophage‐1 antigen (CD11b) (microglial marker), glial fibrillary acidic protein (GFAP) (astrocytic marker), TSPO, 6E10 (Aβ marker), SVA2 (synaptic vesicle marker), MAP2 (microtubule marker) and NeuN (neuronal marker). Animals were culled by isoflurane overdose confirmed by cervical dislocation. The brains were collected, snap frozen using isopentane on dry ice and stored at −80°C. Coronal brain sections (20 μm thick), from the rostral to caudal part of the brain, were taken using a cryostat (Leica CM3050s, Leica Biosystems Nussloch GmbH, Germany) and stored at −80°C. Sections were allowed to defrost and dry at ∼20°C for 20 min and then fixed with 4% paraformaldehyde for 10 min before being washed (6 × 5 min) in phosphate‐buffered saline (PBS) and incubated for 30 min in 2% normal donkey serum and 0.1% Triton X‐100 in PBS to permeabilize and block non‐specific binding. TSPO, 6E10, SV2A and neurogranin immunohistochemistry required an extra step of antigen retrieval done by incubating the slides in 10‐mM citrate buffer at 90°C for 20 min and then washed 2 × 3 min in PBS. Primary antibody incubation was carried out overnight at 4°C with one of the following primary antibodies in 2% normal donkey serum and 0.1% Triton X‐100 in PBS: rat anti‐mouse CD11b (AbD Serotec (MCA711), RRID: AB_321292, 1 : 1000); rabbit anti‐mouse TSPO (Abcam, Cambridge, UK (EPR5384), RRID: AB_10862345, 1 : 250); rabbit anti‐mouse GFAP (DAKO (Z0334), RRID: AB_10013382, 1 : 1000); mouse anti‐human 6E10 amyloid (BioLegend, London, UK (803001), RRID: AB_2564653, 1 : 1000); rabbit anti‐mouse SV2A (Abcam (ab32942), RRID: AB_778192, 1 : 500); chicken anti‐mouse MAP2 (Abcam (ab5392), RRID: AB_2138153, 1 : 1000); rabbit anti‐mouse NeuN (Abcam (ab177487), RRID: AB_2532109, 1 : 500); rabbit anti‐mouse Neurogranin (Abcam (ab23570), RRID: AB_447526, 1 : 500). Following incubation in primary antibody, PBS washes were repeated (3 × 10 min) and incubated with one of the following secondary antibodies was carried out: Alexa Fluor 594 nm Donkey anti‐rat IgG 1 : 500 (for CD11b); Alexa Fluor 488 nm Donkey anti‐rabbit IgG 1 : 500 (for TSPO, GFAP, SV2A); Alexa Fluor 594 nm Donkey anti‐mouse IgG 1 : 500 (for 6E10); Alexa Fluor 488 nm Goat anti‐chicken Double staining was carried out for CD11b+TSPO, CD11b+GFAP and 6E10 + TSPO and to allow the visualization of microglia and astrocytes with TSPO expression and Aβ burden. Double staining was also carried out for MAP2 + NeuN to look at neuronal density.

Images of the hippocampus and cortex were collected between bregma −2.06 mm and −2.30 mm on an Olympus BX51 upright microscope using a 10 × /0.30 or 20 × /0.50 UPlanFLN objective and captured using a Retiga 6000 Color camera through QCapture Pro 7 Software (QImaging Inc., Surrey, Canada). Specific band pass filter sets were used to prevent bleed through from one channel to the next.

### Statistical analysis

No blinding *stricto sensu* was performed; however, animals were identified and recorded only using a unique code number during behavioural tests and image analysis and only identified as TG or WT post‐analysis so that the observers could not know whether the animal being analysed was a WT or a TG. Sample size were calculated to *n* = 9–10 per group using anterior data obtained in our laboratory using the following online tool: https://www.stat.ubc.ca/~rollin/stats/ssize/n2.html (with α = 0.05, β = 0.2, with anticipated mean difference of 8% and SD ~5–8%).

The data were statistically analysed using GraphPad Prism version 5.04. Behavioural data are expressed as mean ± SEM and imaging data are expressed as mean ± SD. Paired *t*‐tests were used to determine differences in exploration in phase 1 of both the NOR and NSR tests. Two‐way analysis of variance (anova) were carried out to test the effect of strain and age on DI, percentage of successful alternation in the Y‐maze, metabolite levels and [^18^F]DPA‐714 uptake in WT and TG mice. Post hoc analysis was carried out using Dunnet's and Sidak's tests. Main effects were considered significant if *p* ≤ 0.05. Interactions were deemed significant if *p* ≤ 0.1. Significance was not adjusted for comparisons of multiple region of interest as those were not compared between them, but if a Bonferroni correction had been applied to the DPA‐714 to account for the number of regions analysed, the appropriate adjusted *p* value would be *p* < 0.017.

## Results

### TG mice display increased cognitive decline

**Figure 2:**
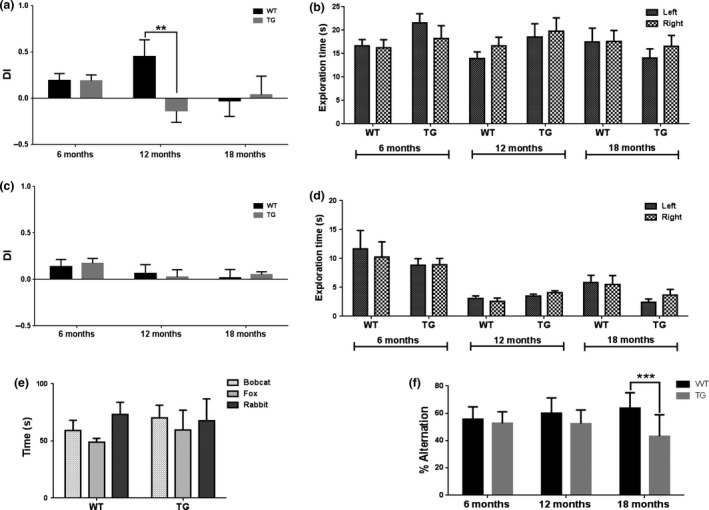
Discrimination index (DI) of exploration of the novel and the familiar object (a) and smell (c) in phase 1 and 2 (WT *n* = 11, TG=15 at 6 months, WT *n* = 6, TG=9 at 12 months, WT *n* = 6, TG = 6 at 18 months). Exploration times of right and left objects in phase 1 of the novel object recognition (NOR) (b) and novel smell recognition (NSR) (d) (*t*‐tests per genotype at each age). Time spent in the presence of predator and non‐predator urine (*n* = 4) (e). Alternation in the Y‐maze (WT *n* = 16, TG = 18 at 6 months, WT = 12, TG = 11 at 12 months, WT = 9, TG = 10 at 18 months) (f). Results are shown as mean ± SEM. Statistical analysis was performed using two‐way anovas and Sidak's multiple comparisons post hoc tests (***p ≤ 0.001).

To test whether short‐term working and recognition memory were affected at different ages, Y‐maze and novel recognition tests were carried out (Fig. 2). No significant differences in DI were seen between WT and TG mice in the NOR at 6 months of age (Fig. 2a). Both groups displayed positive DI results suggesting a preference for the novel and indicating good short‐term memory at this age. However, analysis revealed a significant interaction between gene and age (Fig. 2a, *p* = 0.0225). This effect was because of a significant decrease in cognitive performance in the TG mice compared to the WT mice at 12 months of age as assessed by DI scores (*p* ≤ 0.01). This effect was not replicated at 18 months of age, whereby both groups displayed low DI scores suggesting poor cognitive performance in both WT and TG by this age. No significant differences were seen in the exploration of identical objects in phase 1 of the NOR (Fig. 2b) indicating that there was no side bias in the test.

No significant differences in performance were seen between groups in the NSR (Fig. 2c). At 6 months of age, both WT and TG were able to discriminate between novel and familiar smell, demonstrating good working memory and olfaction. Although no side bias was observed in phase 1 (Fig. 2d), high variation was seen in this test from 12 months of age onwards, therefore the olfactory ability of these mice was tested at this age. In the olfaction test, the behaviour to predator and non‐predator urine was tested. Neither WT nor TG spent significantly more time in the arms containing rabbit urine when compared to fox or bobcat urine (Fig. 2e) indicating olfactory dysfunction from 12 months of age.

TG mice also displayed increased cognitive decline in the Y‐maze test (Fig. 2f). Overall decreased percentages of successful alternation were seen in the TG mice as an effect of gene (*p* = 0.0001). In addition, a significant interaction was observed between gene and age (*p* = 0.0233). This effect did not reveal any significant differences in Y‐maze performance at 6 or 12 months of age. In contrast, at 18 months, a significant decrease in the percentage of alternation was observed in TG mice compared to age‐matched WT mice (*p* ≤ 0.001) indicating accelerated decline in short‐term working memory in TGs at this age.

### [^18^F]DPA‐714 binding significantly increases *in vivo* as a result of AD‐like pathology and age

**Figure 3:**
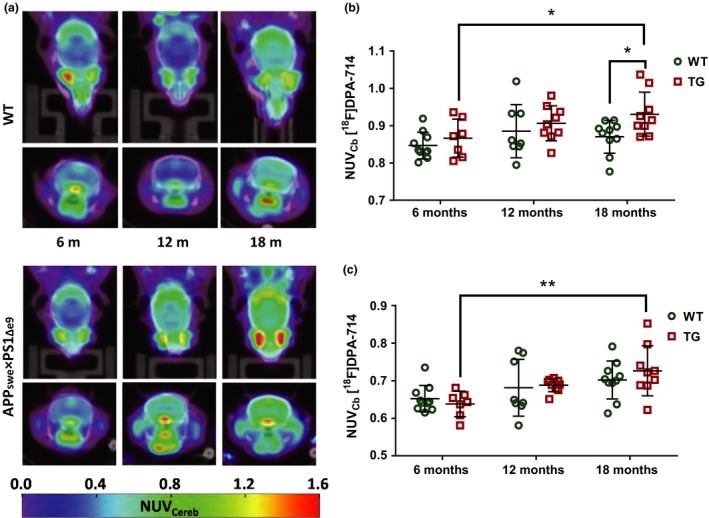
Positron emission tomography (PET) images showing [^18^F]DPA‐714 uptake in WT and APP_swe_×PS1_Δe9_ mice at 6 (WT *n* = 10, TG=7), 12 (WT *n* = 8, TG=9) and 18 months (WT *n* = 10, TG=9) of age (a). NUV_cb_ values in the hippocampus and cortex (b) and other subcortical regions (c). Results are expressed as mean±SD. Statistical analysis was performed using two‐way anova followed by Sidak's and Dunnet's post hoc analysis (*p ≤ 0.05, **p ≤ 0.01).

To assess neuroinflammation differences between APP_swe_×PS1_Δe9_ and WT mice, [^18^F]DPA‐714 NUV_cb_ was compared at 6, 12 and 18 months in the hippocampal+cortical and subcortical ROIs (Fig. 3). Statistical analysis of the cerebellum standard uptake value revealed a significant effect of age only, which the Sidak's post hoc test revealed to be between 6 and 18 months old WT only. No other difference, particularly between WT and TG, could be found in the cerebellum standard uptake value justifying the use of the cerebellum to normalize the uptake values as previously done in this type of study (Serriere *et al*. 2015; Takkinen *et al*. 2016). A two‐way anova revealed a significant effect of gene (*p* = 0.02) and age (*p* = 0.03) on [^18^F]DPA‐714 uptake in the hippocampus and cortex, which resulted in a modest but significant increase in [^18^F]DPA‐714 NUV_cb_ uptake in TG mice at 18 months of age (0.930 ± 0.059) when compared to both age‐matched WT (Fig. 3b, 0.870 ± 0.044; +7%, *p* = 0.04) and 6‐month‐old TG mice (0.866 ± 0.051; +7%, *p* = 0.03). These results suggest that both age and disease increase the neuroinflammatory status of TG mice. An increasing effect was seen with age on [^18^F]DPA‐714 NUV_cb_ values in the other subcortical region (*p* = 0.0008) and a significant increase in [^18^F]DPA‐714 NUV_cb_ uptake was seen in other subcortical regions of 18 months old TG mice (0.638 ± 0.034) when compared to 6‐month‐old TG mice (Fig. 3b, 0.726 ± 0.067; +14%, *p* = 0.002). The same trend was observed in the subcortical regions of WTs but was not significant (Fig. 3c, *p* = 0.058).

### Metabolite profiles of APP_swe_×PS1_Δe9_ and WTs are affected by age

**Figure 4:**
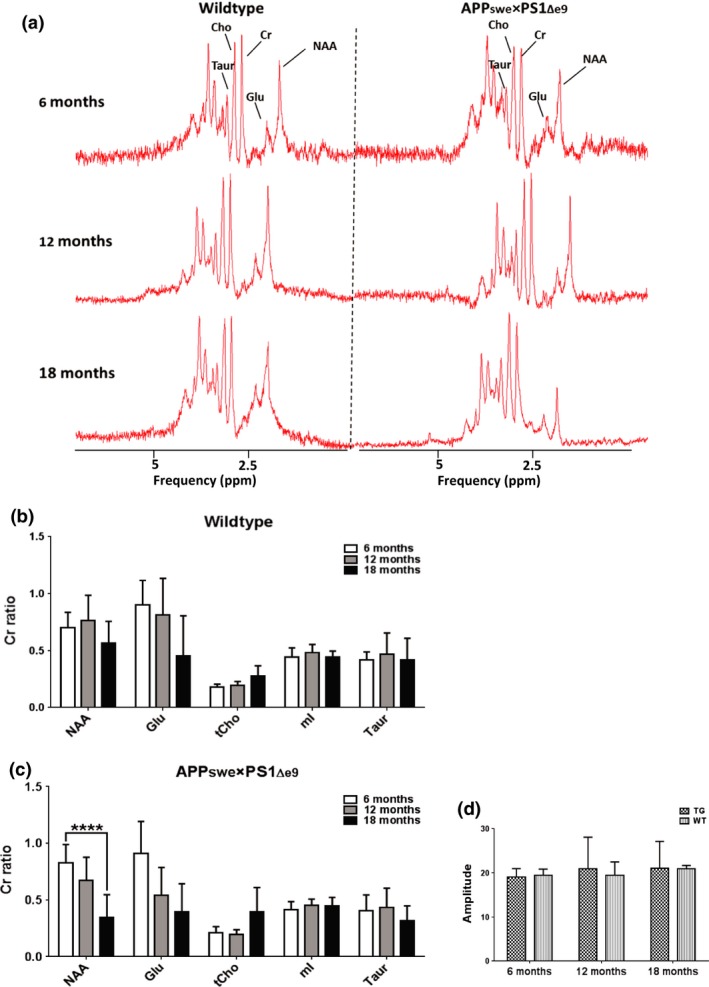
Example spectra from WT and TG mice at 6, 12 and 18 months (a). Metabolites expressed as ratios to creatine in WT (*n* = 6) (b) and TG (*n* = 8) (c) mice at 6, 12 and 18 months of age. No differences were observed in the Cr concentration (mean ± SD) referenced to water (d). Reduced Glu/Cr and increased tCho levels were observed with age in both WT and TG (independently of genotype). Results are shown as mean±SD. Statistical analysis was performed using two‐way anova followed by Dunnet's *post hoc* analysis. (****p ≤ 0.0001).

Single voxel ^1^H MRS was repeated in the same cohort of TG and WT mice at 6, 12 and 18 months of age to investigate changes in metabolite profile. A 3 × 3×3 mm voxel was placed to encompass the hippocampus and the most dorsal part of the thalamus. Example spectra from this region can be seen in Fig. 4a. No significant differences were seen in Cr concentration referenced to water at any age group for either WT or TG mice (Fig. 4d), allowing metabolite data to be expressed as a ratio to Cr. This has been previously reported in this model (Jansen *et al*. 2013); however, it was important to assess this as elevated Cr levels, compared to WT mice, have previously been reported in the double‐mutant APPswe × PS1.M146V (TASTPM) mouse model of AD *in vivo* at an early age (Forster *et al*. 2013) and at a later age analysing brain extracts *in vitro* (Forster *et al*. 2012). No overall significant effect of gene alone was seen on any metabolite in our study. However, a significant effect of age (*p* = 0.0006) and a significant interaction between gene and age (*p* = 0.0866) were observed for NAA. This effect resulted in significantly lower NAA in 18‐month‐old TG mice compared to 6‐month‐old TG mice using multi‐comparisons analysis (−58%, Fig. 4c, *p* ≤ 0.0001). This ageing effect was seen in the WT mice (−20%) but was not statistically significant. Significant ageing effects were also observed on Glu (*p* = 0.0003) and tCho (*p* = 0.0016) levels, resulting in reduced Glu (−53% average across groups from 6 to 18 months) and increased tCho (+71% average across groups from 6 to 18 months) levels with age. No gene effect or gene×genotype interaction was associated with these changes suggesting that these alterations are an effect of normal ageing.

### Immunohistochemistry

**Figure 5:**
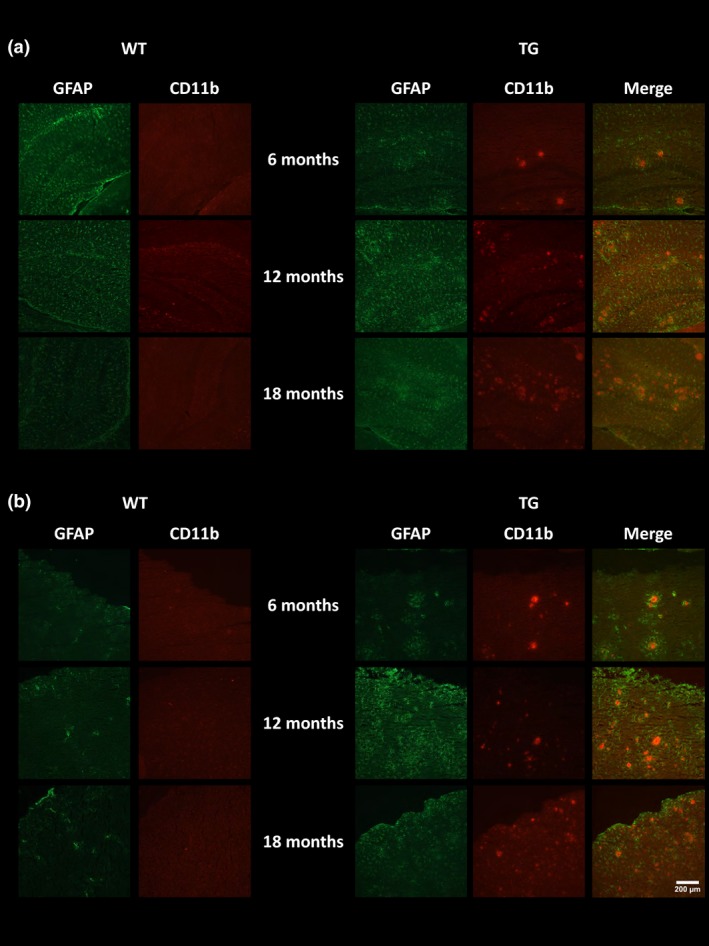
Immunoreactivity of glial fibrillary acidic protein (GFAP) (green) and CD11b (red). Representative images of double staining in the hippocampus (a) and cortex (b) of WT and APP_swe_×PS1_Δe9_ mice at 6, 12 and 18 months of age. Pictures were taken at 10 × magnification between bregma −2.06 mm and −2.30 mm. Scale bar represents 200 μm.

**Figure 6:**
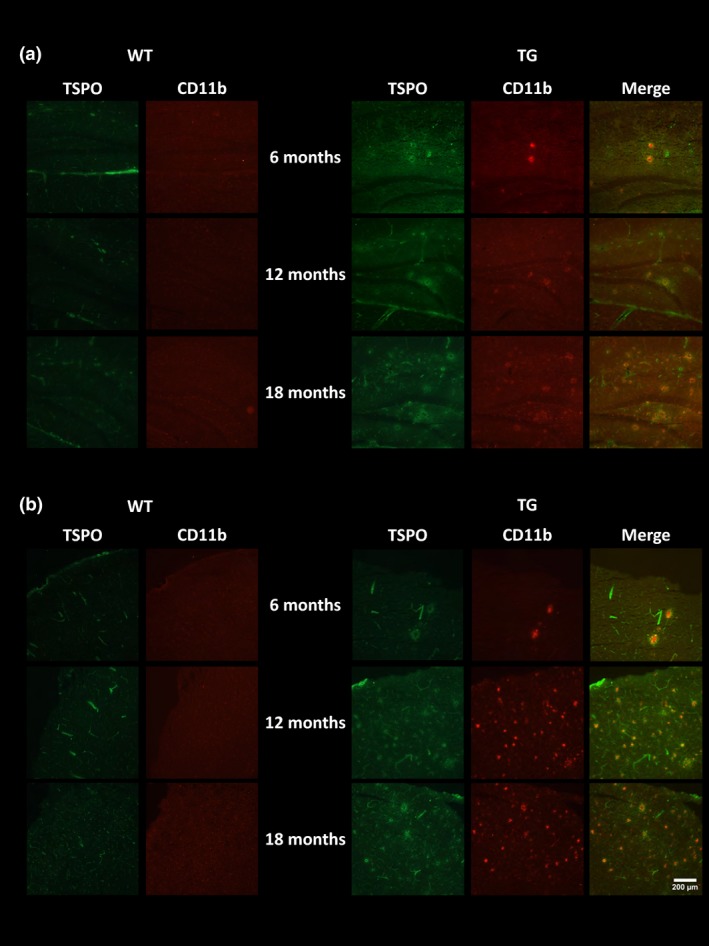
Immunoreactivity of translocator receptor 18 kDa (TSPO) (green) and CD11b (red). Representative images of double staining in the hippocampus (a) and cortex (b) of WT and APP_swe_×PS1_Δe9_ mice at 6, 12 and 18 months of age. Pictures were taken at 10 × magnification between bregma −2.06 mm and −2.30 mm. Scale bar represents 200 μm.

**Figure 7:**
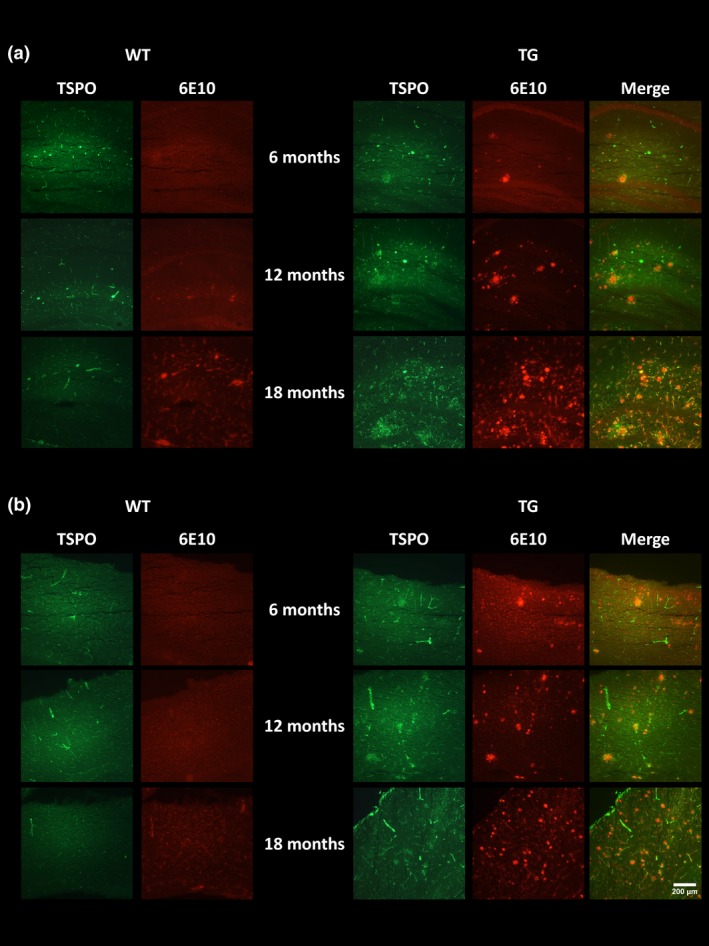
Immunoreactivity of translocator receptor 18 kDa (TSPO) (green) and Aβ (red). Representative images of double staining in the hippocampus (a) and cortex (b) of WT and APP_swe_×PS1_Δe9_ mice at 6, 12 and 18 months of age. Pictures were taken at 10 ×  magnification between bregma −2.06 mm and −2.30 mm. Scale bar represents 200 μm.

**Figure 8:**
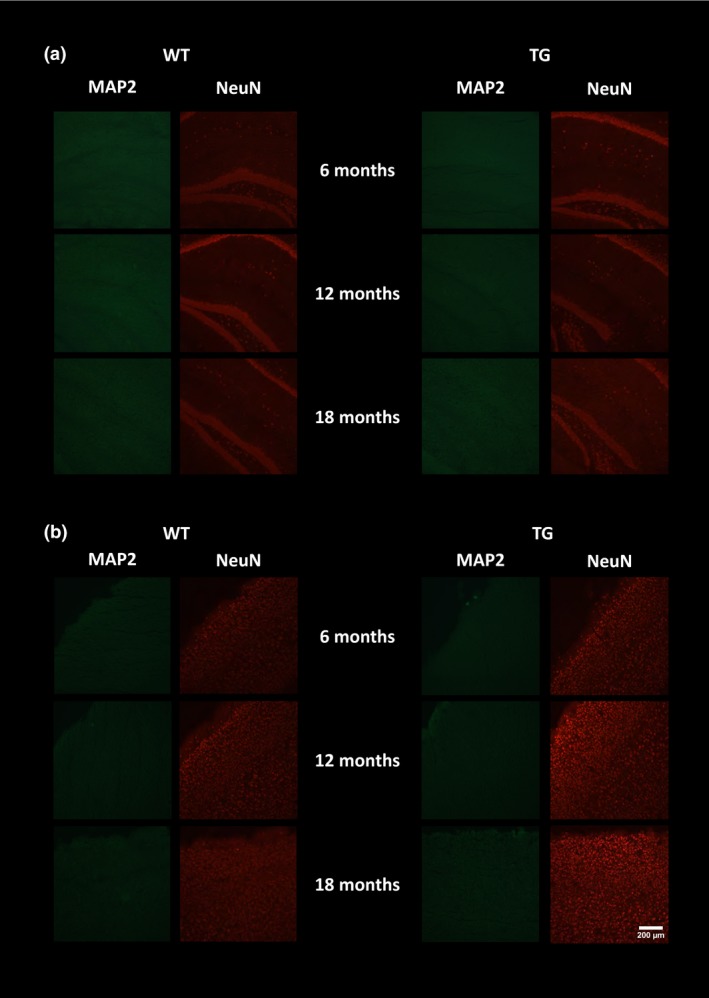
Immunoreactivity of MAP2 (green) and NeuN (red). Representative images of double staining in the hippocampus (a) and cortex (b) of WT and APP_swe_×PS1_Δe9_ mice at 6, 12 and 18 months of age. Pictures were taken at 10 × magnification between bregma −2.06 mm and −2.30 mm. Scale bar represents 200 μm.

**Figure 9:**
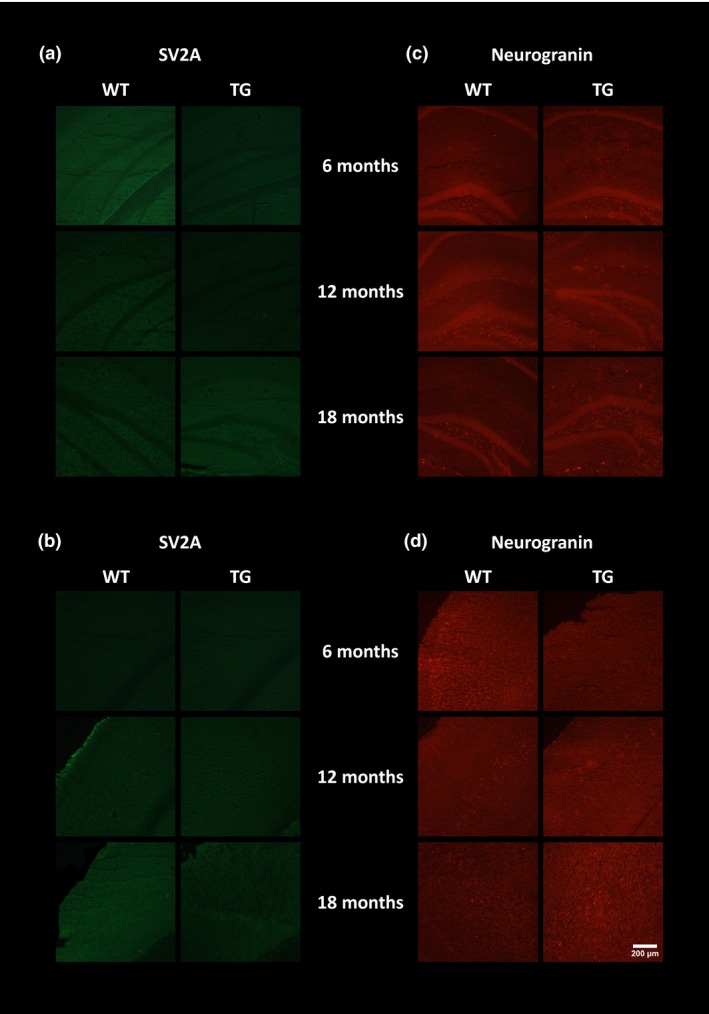
Immunoreactivity of neurogranin (red) and SV2A (green). Representative images of single SVA2 staining in the hippocampus (a) and cortex (b) and neurogranin staining in the hippocampus (c) and cortex (d) of WT and APP_swe_×PS1_Δe9_ mice at 6, 12 and 18 months of age. Pictures were taken at 10 × magnification between bregma −2.06 mm and −2.30 mm. Scale bar represents 200 μm.

To verify the *in vivo* imaging results, e*x vivo* immunohistochemistry was performed to assess the presence of neuroinflammation, Aβ burden and neuronal integrity. GFAP‐ and CD11b‐positive staining were seen in the hippocampus (Fig. 5a) and cortex (Fig. 5b) of TG mice but not WT mice. Low levels of immunostaining were evident at 6 months of age and increased with age in both regions in TG mice. Similarly, double staining for TSPO and CD11b revealed an increase in both proteins with age in the hippocampus (Fig. 6a) and cortex (Fig. 6b) of the TG mice. In the TG mice, there was regional co‐expression of TSPO and CD11b in the hippocampus and cortex, with a modest expression at 6 months of age increasing at 12 months and further at 18 months. In WT animals, TSPO staining was only seen in the vessels and not the parenchyma in both regions. No CD11b staining was evident in the WTs at any age. Immunostaining for TSPO and Aβ pathology (6E10) revealed similar results. An age‐dependent increase was seen in Aβ burden in both the hippocampus (Fig. 7a) and cortex (Fig. 7b) of TG mice only. Aβ staining was sparse at 6 months but increased with age revealing a heavy burden by 18 months. CD11b, TSPO and 6E10 demonstrated good regional co‐expression from 6 to 18 months in the cortex of TG mice, indicating increased microglial activation around Aβ plaques. No Aβ staining was evident in the WT mice at any age. No differences were seen in staining for MAP2, NeuN (Fig. 8a‐b) and SV2A and neurogranin (Figure 9a and b) between TG and WT mice or with age in the hippocampus or cortex indicating that neuronal death could not be detected in this model with this method.

## Discussion

### *In vivo* studies

Cognitive performance in the TG group at 6 months of age was comparable to WT mice and has been previously reported in a variety of memory‐based tests in this (Chen *et al*. 2012; Jansen *et al*. 2013) and other AD models (Nagakura *et al*. 2013; Webster *et al*. 2013). This supports the low levels of Aβ load observed at this age in this and other studies (Jankowsky *et al*. 2004; Garcia‐Alloza *et al*. 2006). TG mice displayed deficits in non‐associative recognition memory and working memory from 12 months, with reduced cognitive performance in the NOR test and Y‐vmaze by 12 and 18 months respectively. Cognitive deficits in these tests have been previously reported in both this (Petrov *et al*. 2015) and other amyloid‐based models of AD (Forster *et al*. 2013; Daniels *et al*. 2016; Martins *et al*. 2017), further supporting the link between pathology burden and cognitive decline. WT mice retained memory later in both tests, displaying increased working memory in the Y‐maze test compared to TG at 18 months. However, no differences in cognition were identified in the NSR test. Both WT and TG were able to discriminate between novel and familiar scents at 6 months of age, demonstrating good working memory and olfaction. However, high variation in both groups was observed in this test from 12 months onwards, therefore we hypothesized that these animals may have altered olfaction by this age, which was proven by the lack of preference of exposure to non‐predator (rabbit) urine over predator (bobcat and fox) urine. An increased amount of time in the arm with non‐predator urine would be expected in mice with intact olfaction; hence the changes observed our NSR study are most likely down to an alteration in olfaction rather than cognitive deficits. These results are, in part, in line with previous publications showing various alterations of olfaction in Tg2576 (Wesson *et al*. 2011) and in APP_swe_×PS1_Δe9_ mice (Yao *et al*. 2016) using different tests and with clinical reports showing that loss of olfaction is common in neurodegenerative diseases and is an early sign of AD (Mesholam *et al*. 1998). We, however, also observed a loss of olfaction in our WT mice. Similarly, some previous studies reported early cognitive dysfunction prior to pathology development using different behavioural assessments such as the Morris water maze (Zhang *et al*. 2012). Here, we report decline in memory with age in APP_swe_×PS1_Δe9_ mice, which is in support of our working hypothesis that cognitive performance decreases with age at a faster rate in the TG mice compared to the WT.

It has been hypothesized that neuroinflammation precedes clinical manifestation of cognitive dysfunction in AD. In this study, we observed the presence of neuroinflammation in TG mice via immunohistochemistry (CD11b and GFAP) from 6 months which increased with age. However, increased neuroinflammation and altered metabolite profile, assessed via *in vivo* PET and MRS, respectively, occurred after the emergence of cognitive decline in this animal model.

Increased [^18^F]DPA‐714 NUV_cb_ was found in the hippocampal and cortical region of old TG mice when compared to both age‐matched WT mice and young TG mice, suggesting that increased neuroinflammation in hippocampal and cortical region is driven by the presence of AD pathology in TG mice. This increase was only statistically significant at 18 months of age despite increased Aβ, GFAP and CD11b immunostaining evident from 12 months of age. Although a significant increase is also seen with age in [^18^F]DPA‐714 uptake in the other subcortical regions, no significant differences were found between genotypes. A trend towards significance was also observed in the WTs in the subcortical ROI. As these regions have little to no amyloid burden, these data suggest that neuroinflammation in these regions is increased mostly as a result of normal ageing and independently of amyloid pathology. It is, however, notable that Yokokura *et al*. (2017) recently reported increased uptake in the thalamus of AD and elderly patients using [^11^C]DPA‐713. These results suggest that neuroinflammation is present prior to cognitive dysfunction; however, current *in vivo* methods are not sensitive enough (because of a combination of tracer sensitivity, resolution capabilities of small animal PET and non‐specific relevance of the mI signal) to detect the more subtle inflammatory changes in the earlier stages of disease that are achievable via *ex vivo* methods.

Our data are in agreement with previous reports in mouse models using [^18^F]DPA‐714. Serriere *et al*. (2015) investigated [^18^F]DPA‐714 uptake in the same mouse model at 6, 9, 12, 15 and 19 months of age. NUV_Cb_ were increased in the cortex of TG mice at 12 and 19 months (but not 15 months of age) and in the hippocampus at 19 months of age. Increased cortical [^18^F]DPA‐714 NUV_cb_ has also been reported in the APP/PS1‐21 mouse from 6 months of age, with hippocampal increases emerging from 12 months of age (Takkinen *et al*. 2016). In our analysis methods, we pooled cortical and hippocampal areas as one region because of AD pathology present in both regions and because the size of the pooled ROI is more realistically compatible with the resolution of PET imaging in preclinical scanners (~1–1.6 mm). Therefore, by pooling the hippocampus and cortex into a single ROI, we may have masked the possible differential time‐effect between each region, and hidden the earlier increase in neuroinflammation in the cortex. However, such difference would be small as our immunohistochemistry results show only a slightly stronger signal in CD11b and TSPO in the cortex than in the hippocampus in this model at 6 and 12 months of age. On the other hand, it can also be argued that thin ROIs including only the cortical hippocampal areas (~1.5 mm thick) are far more subject to partial volume effects, which could consequently have biased the quantification in previous studies. A significant effect of both age and gene on TSPO PET has been also recently reported in the same model using [^18^F]GE‐180 (Liu *et al*. 2015). Increased [^18^F]GE‐180 uptake was seen in the hippocampus of old TGs compared to age‐matched WTs and young TG and WT mice. In addition, increased [^18^F]GE‐180 uptake was also seen in the whole brain of old TGs compared to young TGs. This age effect was replicated in WT mice with significant increases in uptake from young to old mice in both hippocampal and whole brain, implicating both normal ageing and AD pathology on neuroinflammatory status. Increased neuroinflammation has also been observed as an effect of ageing in both humans (Gulyas *et al*. 2011; Kumar *et al*. 2012; Yokokura *et al*. 2017) and in the WTs of other AD models (Brendel *et al*. 2016). Altogether, our results further support previous findings that age can significantly alter microglial responses, which are modified further in the presence of neurodegenerative diseases such as AD. Increases in TSPO expression have been found in other models of AD (Ji *et al*. 2008; James *et al*. 2015; Brendel *et al*. 2016; Mirzaei *et al*. 2016) and in human AD (Forlenza *et al*. 2009; Swardfager *et al*. 2010; Rubio‐Perez and Morillas‐Ruiz 2012; Varnum and Ikezu 2012), indicating that elevated neuroinflammation is a consistent characteristic of this disease. The modest increases and the relative overlap between WT and TG mice demonstrated by the present data and others’ are also in agreement with the PET quantification in AD patient versus healthy controls which generally reports non‐significant (Varrone *et al*. 2013) or small to more substantial increases (+10−35%) (Edison *et al*. 2008; Okello *et al*. 2009; Schuitemaker *et al*. 2013; Varrone *et al*. 2015; Hamelin *et al*. 2016) in neuroinflammation. This demonstrates that (i) measuring neuroinflammation *in vivo* in AD is challenging because the amplitudes of changes are overall modest when compared with the changes induced by ageing only and (ii) that, at least from this point of view, animal models are actually reflecting the clinical situation quite well. Finally, a recent study by Owen *et al*. (2017) suggests that, at least in human, TSPO expression may reflect microglia density rather than microglia phenotype. Although obtained from a purely *in vitro* setting in which cells may behave differently than in their *in vivo* environment, these observations taken together with the known TSPO expression by endothelial cells may explain the inconsistencies between imaging studies and the difficulties encountered over the years to truly assess microglial activation in AD patients using TSPO PET.

In contrast to increased neuroinflammation assessed by PET and immunohistochemistry, no alterations in mI levels were observed between TG and WT mice in this study. This lack of agreement between neuroinflammatory status and mI expression questions the role of mI as a putative marker for gliosis with the specific biological significance of mI needing further investigation to be truly elucidated. In support of this, reported mI level alterations are inconsistent in this model, with both increased and stable levels reported (Dedeoglu *et al*. 2004; von Kienlin *et al*. 2005; Jansen *et al*. 2013). Moreover, in a clinical study, mI was found to be associated with amyloid pathology and not neuroinflammation (Murray *et al*. 2014), therefore it is possible that mI levels represent more closely amyloid load than neuroinflammation. On the other hand, we have extensive amyloid burden in this model without seeing mI alterations. These results are in contrast to many other reports demonstrating increased mI levels in clinical AD (Kantarci *et al*. 2007; Shinno *et al*. 2007; Foy *et al*. 2011; Shiino *et al*. 2012; Murray *et al*. 2014) and AD models (Marjanska *et al*. 2005, 2014; Jack *et al*. 2007; Oberg *et al*. 2008; Chen *et al*. 2009, 2012; Choi *et al*. 2010, 2014; Yang *et al*. 2011; Forster *et al*. 2013). Chen *et al*. (2009) reported significantly higher mI/Cr levels in the APP_swe_×PS1_Δe9_ compared to WT mice as early as 3 months of age. However, the voxel used in this study included cortical as well as hippocampal tissue. Here, we report the appearance of inflammation and plaques in cortical regions prior to and more frequently than in hippocampal regions, so the discrepancies between studies might be because of differences in size and location of the voxel used as well as the age studied.

Although no qualitative changes were observable in neuronal markers (MAP2 and NeuN) using immunohistochemistry, a significant effect of ageing and a significant genotype×age interaction was observed on NAA levels, resulting in a ‐58% decrease in NAA/Cr in 18‐month‐old TG mice when compared to 6‐month‐old TG. This effect was not mirrored in the WT mice and suggests that decreased NAA levels, although influenced by both age and gene, are slightly more pronounced in TG mice. This underlines the importance of understanding the pattern of normal ageing within brain metabolites or other potential biomarkers and the possible confounding effects of age.

Chen *et al*. (2009) reported a small but significant decrease (−11% change, 1.16 ± 0.07 in WT vs. 1.03 ± 0.06 in TG) in NAA/Cr from 5 months of age. In contrast, we report NAA alterations post‐cognitive decline and is in line with the results from Xu *et al*. (2010) in the same animal model. Xu *et al*. (2010) found a significant decrease in hippocampal NAA/Cr in 16‐month‐APP_swe_×PS1_Δe9_ TG mice compared to younger TG mice, which was not seen in WT mice. Similar decreases in NAA/Cr associated with age and AD pathology have been reported in other models of AD, at varying ages (Marjanska *et al*. 2005; Jack *et al*. 2007; Oberg *et al*. 2008; Choi *et al*. 2010, 2014; Xu *et al*. 2010; Forster *et al*. 2013; Jansen *et al*. 2013). We also report age to have a significant effect on Glu and tCho levels without an effect of genotype effect or an interaction genotype × age, in which Glu levels were decreased and tCho levels increased in both WT and TG mice. Similarly, specific reduced Glu and increased tCho levels have been reported in mouse models of AD (Dedeoglu *et al*. 2004; von Kienlin *et al*. 2005; Marjanska *et al*. 2005; Jack *et al*. 2007; Oberg *et al*. 2008; Choi *et al*. 2010; Chen *et al*. 2012; Esteras *et al*. 2012) and in the clinical situation (Hattori *et al*. 2002; Kantarci 2007; Griffith *et al*. 2008; Foy *et al*. 2011; Shiino *et al*. 2012) versus WT or healthy controls respectively. This result suggests that these effects are a result of normal ageing in this strain.

### Ex vivo immunohistochemistry

To verify the *in vivo* imaging results, e*x vivo* immunohistochemistry was performed to assess the presence of microglia, astrocytes, TSPO expression and Aβ pathology. Aβ pathology was sparse in young TG mice with increasing burden evident by 12 months and abundant plaques by 18 months. This level of Aβ burden is in line with many previous reports in this model (Jankowsky *et al*. 2004; Garcia‐Alloza *et al*. 2006; van Groen *et al*. 2006). Similarly, low levels of CD11b, GFAP and TSPO staining were evident in the hippocampus and cortex of TG mice at 6 months, which increased with age. Co‐localization of CD11b and TSPO was observed in the cortex and the hippocampus, confirming that in this model, TSPO is mostly expressed by microglia. Similarly, TSPO and Aβ co‐localization was observed in the cortex of TG mice, indicating increased glial activation around Aβ plaques. Similarly, strong astrogliosis was detected around microglial cells which were found only around Aβ plaques supporting the presence of both astrocytes and microglia around Aβ plaques. Microglial cells are known to surround plaques in human AD and have been shown to co‐localize with pathology in AD models further supporting the role of neuroinflammation in AD. In contrast to our results, Ji *et al*. (2008)*,* did not find co‐expression of CD11b and TSPO in the APP23 mouse model, but found co‐localization of GFAP and TSPO which were in close proximity to amyloid plaque staining. However, the opposite was identified in a tau model, with CD11b but not GFAP‐positive cells expressing TSPO. These results suggest that different forms of tau and amyloid pathology may alter TSPO expression differently *in vivo* (Jansen *et al*. 2013). This may be because of potential differences in type of amyloid, aggregation properties, expression levels and age of pathology emergence between different models of AD, which may differently affect the glial response (Stalder *et al*. 1999; Xiong *et al*. 2011; Huang *et al*. 2016). In contrast, TSPO expression was only visible in the vessels in WT animals which are in agreement with previous report demonstrating the expression of TSPO in vessels (Turkheimer *et al*. 2007).

We did not identify any striking reductions in neuronal, synaptic vesicle or microtubule markers in this study. This is in line with previous reports (Jansen *et al*. 2013) suggesting that APP mutations are not sufficient to cause neuronal loss that is observed in human AD and models with tau abnormalities but in contrast with Huang *et al*. (2016) who reported a decrease (~20%) in NeuN staining and hippocampal atrophy (~20–30%). On the other hand, amyloid pathology induced cognitive decline and resulted in accelerated NAA loss with age in TG compared to WT mice. This lack of congruency could be explained by many factors such as assessing the wrong neuronal/synaptic markers, changes being too small or regionally specific or as a result of changes in certain neuronal populations (e.g. cholinergic) to be detectable using the methods and markers used here.

## Conclusion

It has become evident that Alzheimer's disease is a complex multifactorial disease in which neuroinflammation plays a pivotal role. In support of this, we report increased neuroinflammation in the form of increased [^18^F]DPA‐714 uptake, confirmed through the use of immunohistochemistry, and reduced neuronal function in the form of accelerated NAA reductions in the APP_swe_×PS1_Δe9_ similar to those measured in AD patients_._ We here show that TSPO PET can detect changes in neuroinflammation in this mouse model, however, not as early as detected using *ex vivo* techniques. This suggests that, although TSPO PET is a viable imaging technique to study AD in animal models, the current restrictions because of resolution and brain size in mice hamper earlier detection. Moving to larger species such as rats may address this. Overall, these results support the role of neuroinflammation in the pathogenesis of AD and the potential use of metabolite alteration to monitor disease progression or response to treatments.

## Supporting information


**Figure S1.** Whole brain was segmented into the hippocampus and cortex (**a**), other subcortical (**b**) and cerebellum (**c**) regions of interest for PET quantification using a modified version of the Waxholm space template (Johnson *et al*. 2010). The MRS voxel was centred at bregma ‐2.30 mm according to the Paxinos mouse brain atlas and encompassed hippocampal and thalamic regions (**d**). Example of an MRS spectrum i*n vivo* with the main metabolites highlighted (**e**). AAP‐amino acid proton, mI‐myo‐Inositol. Tau‐taurine, Cho‐choline containing compounds, Cre‐ creatine+phosphocreatine, Glu‐ glutamate, GABA‐ γ‐aminobutyric acid, NAA‐ N‐acetylaspartate, Lipid MMS‐ lipid and macromolecules.
**Figure S2.** [^18^F]DPA‐714 standard uptake value in the cerebellum of WT and APPswe×PS1Δe9 mice at 6 (WT *n* = 10, TG=7), 12 (WT *n* = 8, TG=9) and 18 months (WT *n* = 10, TG=9) of age. Results are expressed as mean±SD. Statistical analysis was performed using two‐way ANOVA followed by Sidak's post hoc analysis (*p ≤ 0.05).
**Figure S3.** Immunoreactivity of GFAP (green) and CD11b (red). Representative images of double staining in the hippocampus (a) and cortex (b) of WT and APP_swe_×PS1_Δe9_ mice at 6, 12 and 18 months of age. Pictures were taken at 20 ×  magnification between bregma −2.06 mm and −2.30 mm. Scale bar represents 200 μm.
**Figure S4**. Immunoreactivity of TSPO (green) and CD11b (red). Representative images of double staining in the hippocampus (a) and cortex (b) of WT and APPswe×PS1Δe9 mice at 6, 12 and 18 months of age. Pictures were taken at 20 ×  magnification between bregma −2.06 mm and −2.30 mm. Scale bar represents 200 μm.
**Figure S5.** Immunoreactivity of MAP2 (green) and NeuN (red). Representative images of double staining in the hippocampus (a) and cortex (b) of WT and APP_swe_×PS1_Δe9_ mice at 6, 12 and 18 months of age. Pictures were taken at **20 × ** magnification between bregma −2.06 mm and −2.30 mm. Scale bar represents 200 μm.

**Data S1.** Supplementary materials.
