# Supplementary material for: Longitudinal investigation of neuroinflammation and metabolite profiles in the APP swe×PS1Δe9 transgenic mouse model of Alzheimer's disease
Source: J Neurochem. 2017 Dec 15;144(3):318–35. doi: 10.1111/jnc.14251 (PMC5846890; doi:10.1111/jnc.14251)
Supplement: Supplementary file 1 — Figure S1. Whole brain was segmented into the hippocampus and cortex (a), other subcortical (b) and cerebellum (c) regions of interest for PET quantification using a modified version of the Waxholm space template (Johnson et al. 2010). The MRS voxel was centred at bregma ‐2.30 mm according to the Paxinos mouse brain atlas and encompassed hippocampal and thalamic regions (d). Example of an MRS spectrum in vivo with the main metabolites highlighted (e). AAP‐amino acid proton, mI‐myo‐Inositol. Tau‐taurine, Cho‐choline containing compounds, Cre‐ creatine+phosphocreatine, Glu‐ glutamate, GABA‐ γ‐aminobutyric acid, NAA‐ N‐acetylaspartate, Lipid MMS‐ lipid and macromolecules. Figure S2. [18F]DPA‐714 standard uptake value in the cerebellum of WT and APPswe×PS1Δe9 mice at 6 (WT n = 10, TG=7), 12 (WT n = 8, TG=9) and 18 months (WT n = 10, TG=9) of age. Results are expressed as mean±SD. Statistical analysis was performed using two‐way ANOVA followed by Sidak's post hoc analysis (*p ≤ 0.05). Figure S3. Immunoreactivity of GFAP (green) and CD11b (red). Representative images of double staining in the hippocampus (a) and cortex (b) of WT and APPswe×PS1Δe9 mice at 6, 12 and 18 months of age. Pictures were taken at 20 × magnification between bregma −2.06 mm and −2.30 mm. Scale bar represents 200 μm. Figure S4. Immunoreactivity of TSPO (green) and CD11b (red). Representative images of double staining in the hippocampus (a) and cortex (b) of WT and APPswe×PS1Δe9 mice at 6, 12 and 18 months of age. Pictures were taken at 20 × magnification between bregma −2.06 mm and −2.30 mm. Scale bar represents 200 μm. Figure S5. Immunoreactivity of MAP2 (green) and NeuN (red). Representative images of double staining in the hippocampus (a) and cortex (b) of WT and APPswe×PS1Δe9 mice at 6, 12 and 18 months of age. Pictures were taken at 20 × magnification between bregma −2.06 mm and −2.30 mm. Scale bar represents 200 μm. [file JNC-144-318-s001.pdf]

Longitudinal investigation 1 of neuroinflammation and metabolite profiles in the APP_{swe}×$PS1_{\Delta e9}$ transgenic mouse model of Alzheimer's disease.

Chaney A.[1,2]*, Bauer M.[3†], Bochicchio D.[2†], Smigova A.[1,2], Kassiou M.[4], Davies K.E.[1]4 , Williams S.R.[1], Boutin H.[1,2].

[1] Centre for Imaging Science, Faculty of Biology, Medicine and Health and Manchester Academic Health Sciences Centre University of Manchester, Manchester, M13 9PT, UK
[2] Wolfson Molecular Imaging Centre, Faculty of Biology, Medicine and Health and Manchester Academic Health Sciences Centre, University of Manchester, Manchester, M20 3LJ, UK.
[3] Medical University Vienna, Department of Clinical Pharmacology, Waehringer Guertel 18-20, 1090 Vienna, Austria.
[4] School of Chemistry, University of Sydney, NSW 2006 Australia.

† Contributed equally to the work

* Current address: 3165 Porter Drive, Stanford University, Palo Alto, CA, 94304.

Corresponding Author:
Dr Hervé Boutin,
Wolfson Molecular Imaging Centre,
27 Palatine Road, Manchester, M20 3LJ, UK
Email: herve.boutin@manchester.ac.uk
Tel: +44 161 275 0078
Fax: +44 161 275 0003

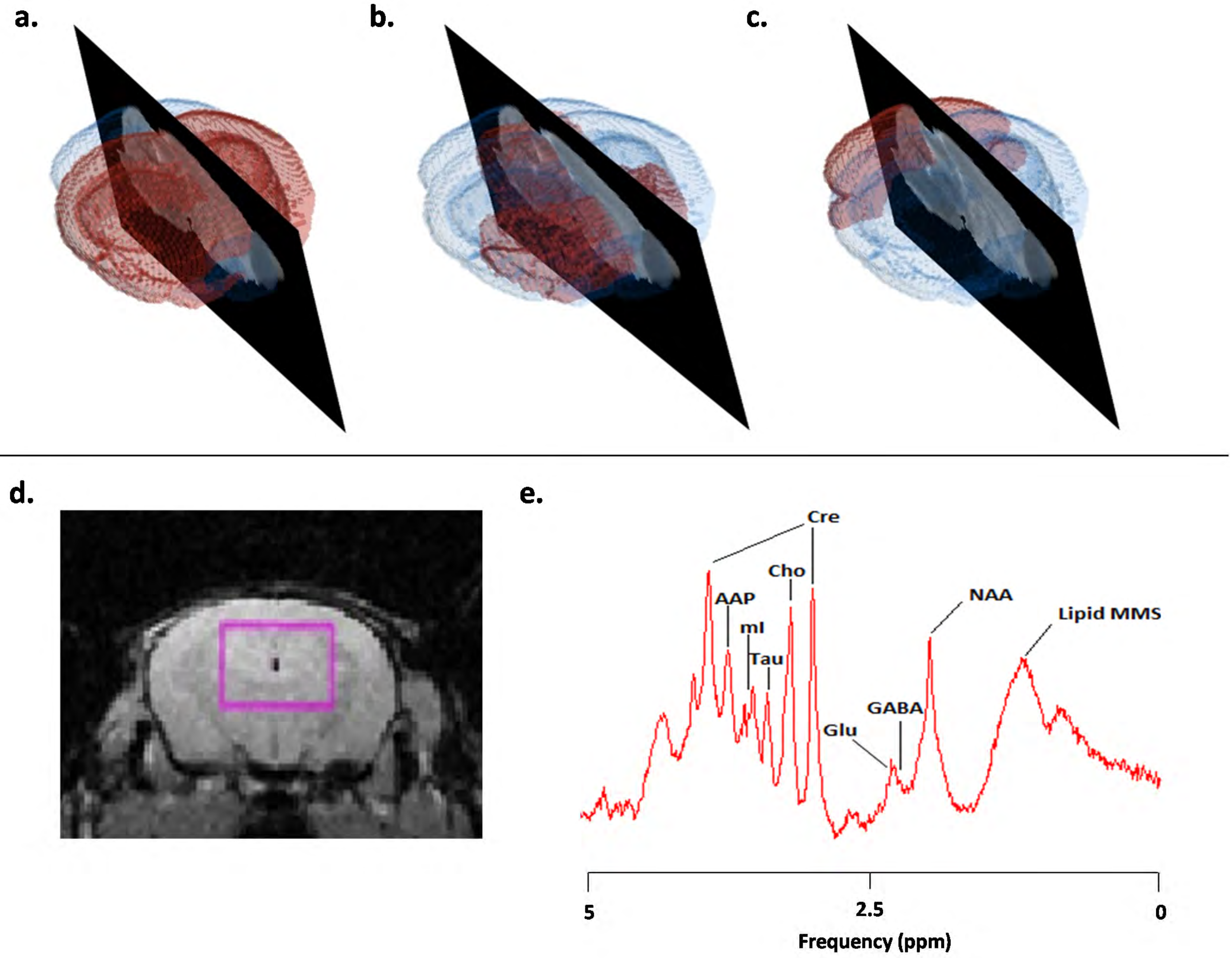

Supplementary figure 1: Whole brain was segmented into the hippocampus and cortex (**a**), other subcortical (**b**) and cerebellum (**c**) regions of interest for PET quantification using a modified version of the Waxholm space template (Johnson et al. 2010). The MRS voxel was centred at bregma -2.30mm according to the Paxinos mouse brain atlas and encompassed hippocampal and thalamic regions (**d**). Example of an MRS spectrum i*n vivo* with the main metabolites highlighted (**e**). AAP-amino acid proton, mI-myo-Inositol. Tau-taurine, Cho-choline containing compounds, Cre- creatine+phosphocreatine, Glu- glutamate, GABA- γ-aminobutyric acid, NAA- N-acetylaspartate, Lipid MMS- lipid and macromolecules.

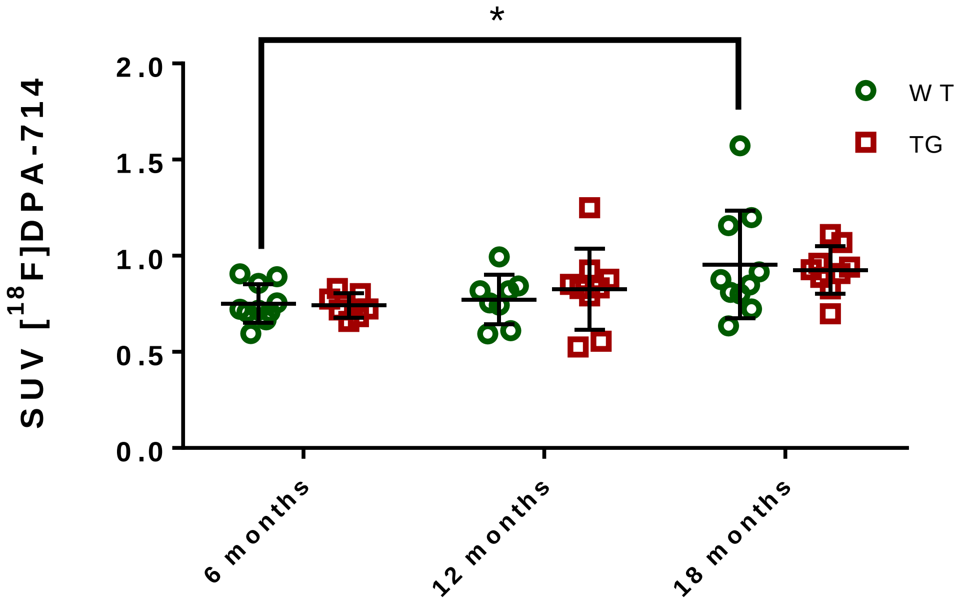

Supplementary Figure 2: [^{18}F]DPA-714 standard uptake value in the cerebellum of WT and APP_{swe}×$PS1_{\Delta e9}$ mice at 6 (WT n=10, TG=7), 12 (WT n=8, TG=9) and 18 months (WT n=10, TG=9) of age. Results are expressed as mean±SD. Statistical analysis was performed using two-way ANOVA followed by Sidak's post-hoc analysis (*p≤0.05).

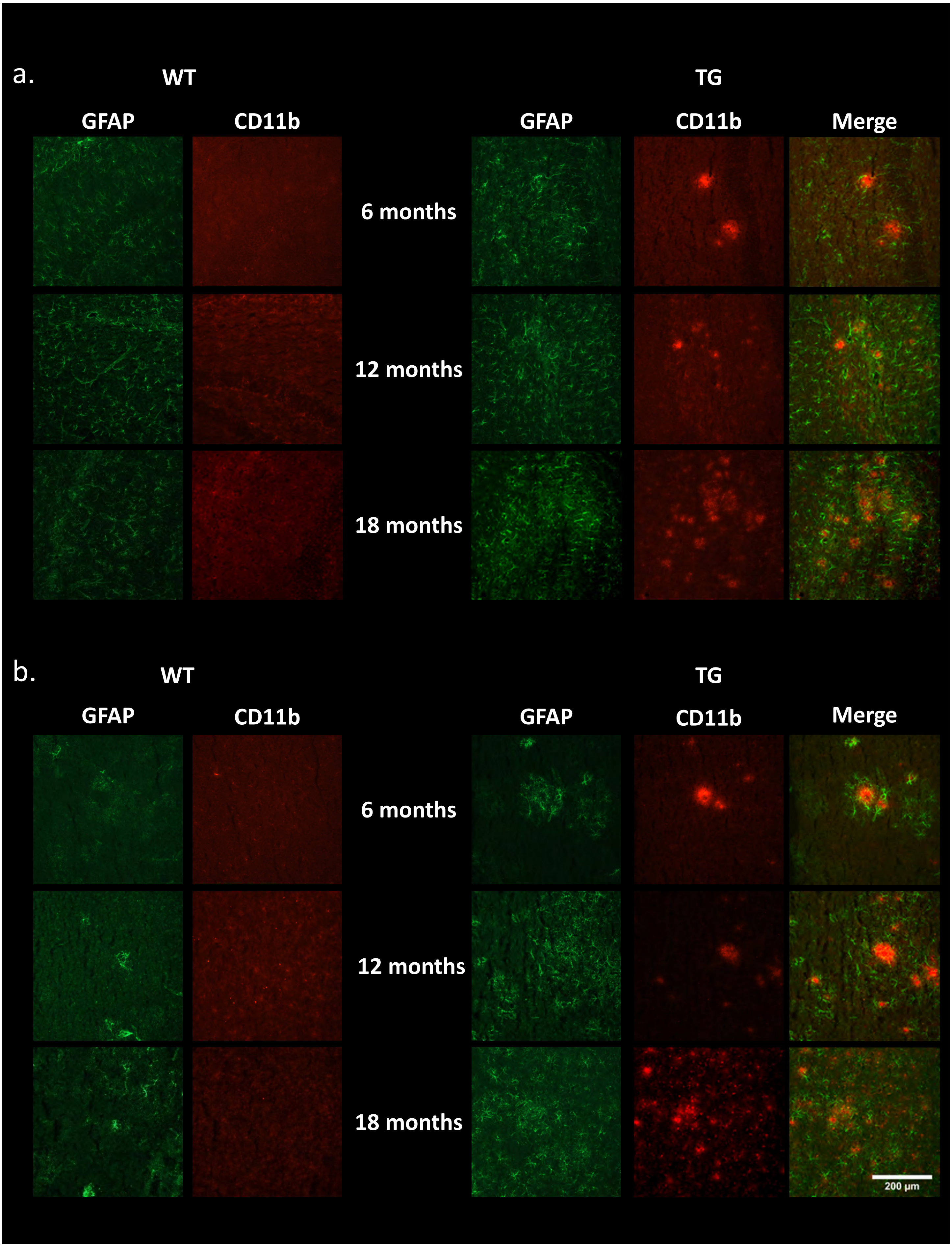

Supplementary figure 3: Immunoreactivity of GFAP (green) and CD11b (red). Representative images of double staining in the hippocampus (**a**) and cortex (**b**) of WT and APP_{swe}×$PS1_{\Delta e9}$ mice at 6, 12 and 18 months of age. Pictures were taken at **20×** magnification between bregma -2.06mm and -2.30mm. Scale bar represents 200µm.

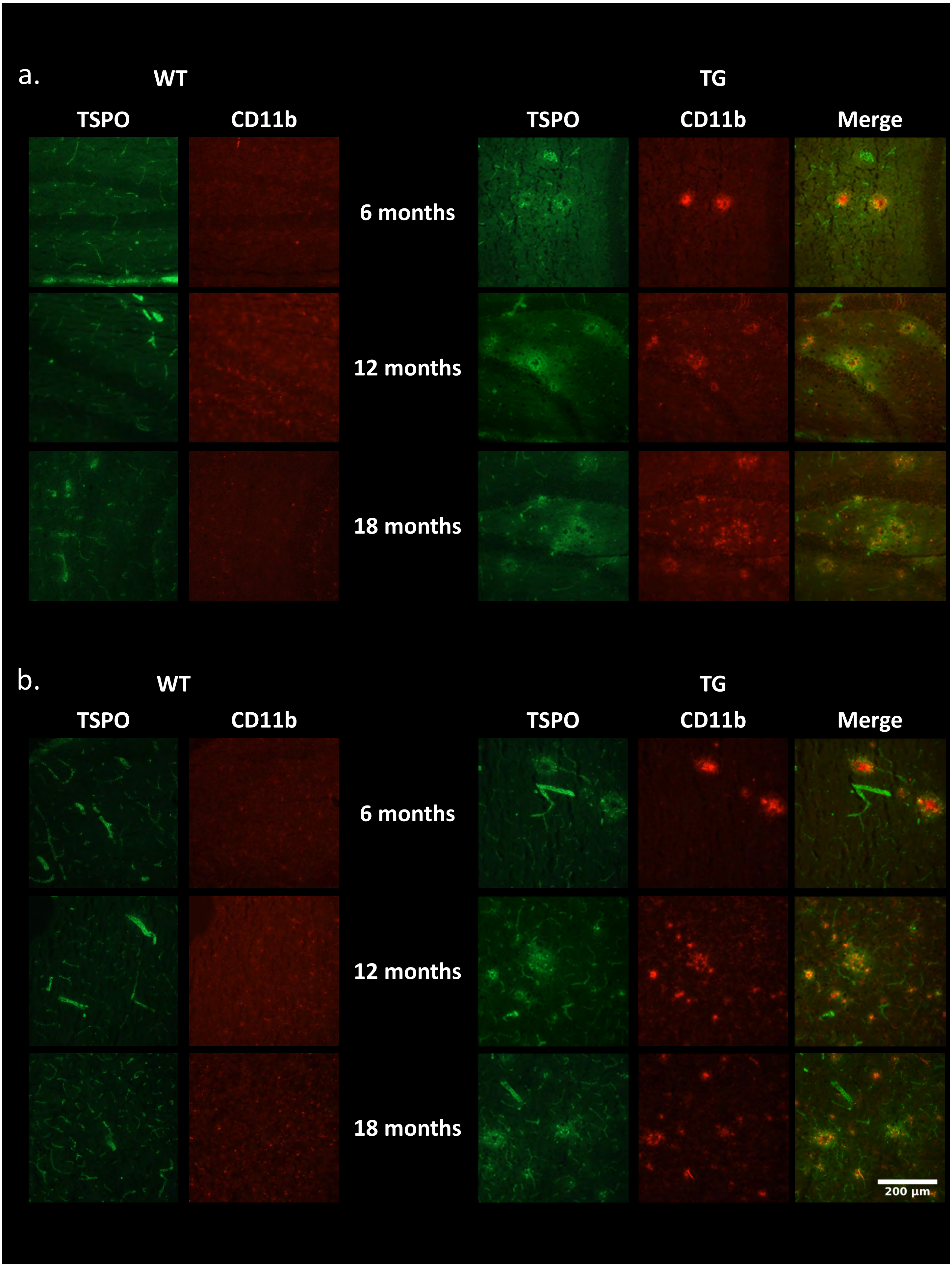

Supplementary figure 4: Immunoreactivity of TSPO (green) and CD11b (red). Representative images of double staining in the hippocampus (a) and cortex (b) of WT and APPswe×PS1Δe9 mice at 6, 12 and 18 months of age. Pictures were taken at **20×** magnification between bregma -2.06mm and -2.30mm. Scale bar represents 200µm.

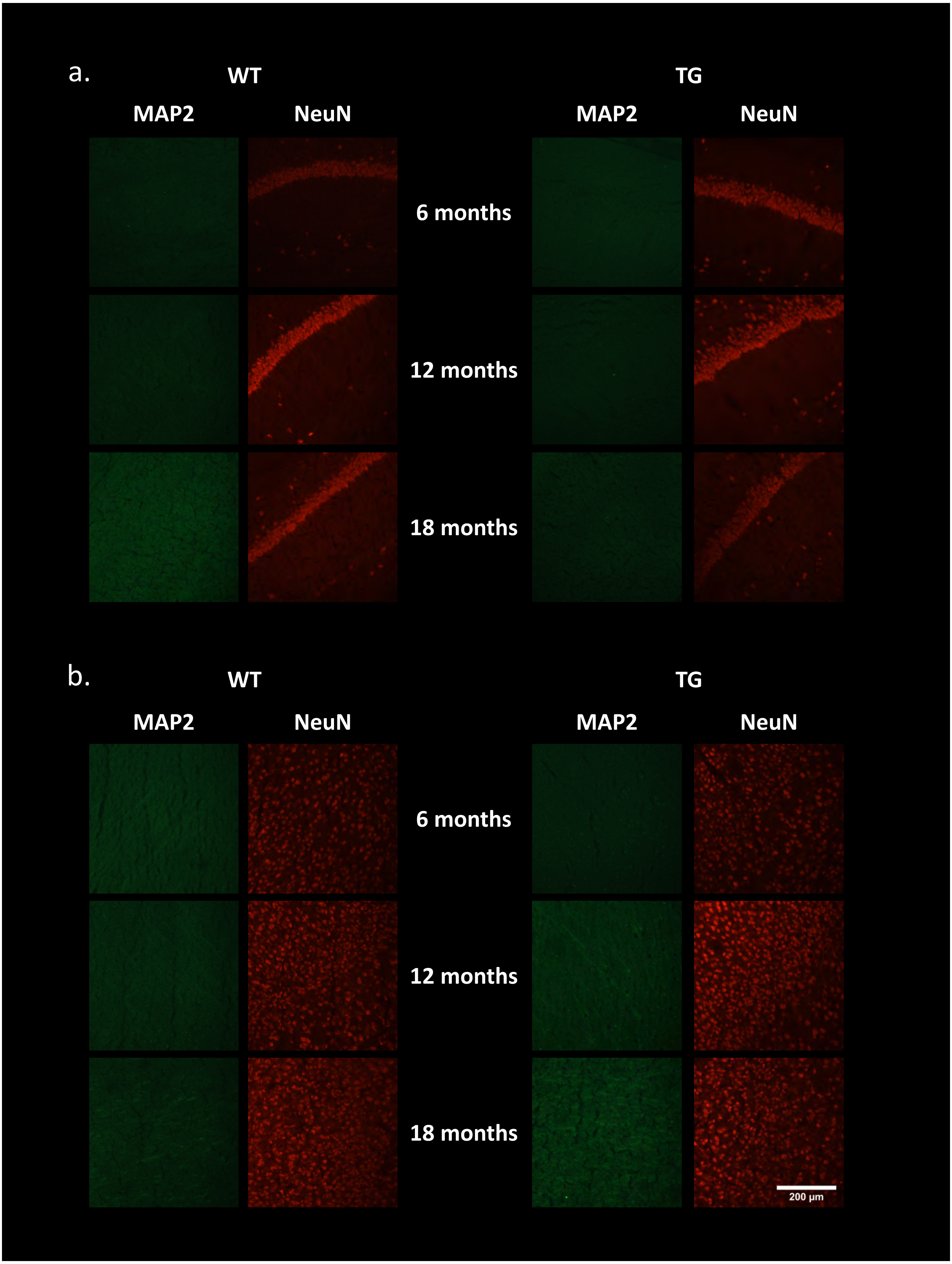

Supplementary figure 5: Immunoreactivity of MAP2 (green) and NeuN (red). Representative images of double staining in the hippocampus (**a**) and cortex (**b**) of WT and $APP_{swe} \times PS1_{\Delta e9}$ mice at 6, 12 and 18 months of age. Pictures were taken at **20×** magnification between bregma -2.06mm and -2.30mm. Scale bar represents 200µm.
