# Supplementary material for: Longitudinal investigation of neuroinflammation and metabolite profiles in the APP swe×PS1Δe9 transgenic mouse model of Alzheimer's disease
Source: J Neurochem. 2017 Dec 15;144(3):318–35. doi: 10.1111/jnc.14251 (PMC5846890; doi:10.1111/jnc.14251)
Supplement: Supplementary file 2 — Data S1. Supplementary materials. [file JNC-144-318-s002.docx]

# Methods

## PET imaging

CT scans were performed prior to PET acquisition to obtain the attenuation correction map. The time coincidence window was set to 3.432 ns and levels of energy discrimination to 350 keV and 650 keV. List mode data from emission scans were histogrammed into 16 dynamic frames (5×1 min; 5×2 min; 3×5 min and 3×10 min) and emission sinograms were normalised, corrected for attenuation, scattering and radioactivity decay and reconstructed using an OSEM3D protocol (16 subsets and 4 iterations) into images of dimensions 128 (transaxially) ×159 (axially) with 0.776×0.776×0.796 mm voxels. The PET images segmented using the local means analysis method and the organ mean time activity curves were corrected for partial volume effect as previously described (Boutin et al. 2013, Maroy et al. 2008, Maroy et al. 2010). The correction method combined the geometric transfer matrix (GTM) method and the regions of interest (ROI)-opt method.

## Immunohistochemistry

Sections were allowed to defrost and dry at room temperature for 20min and then fixed with 4% paraformaldehyde for 10min before being washed (6×5min) in phosphate buffered saline (PBS) and incubated for 30 minutes in 2% normal donkey serum and 0.1% Triton X-100 in PBS to permeabilize and block non-specific binding. TSPO, 6E10, SV2A and neurogranin immunohistochemistry required an extra step of antigen retrieval done by incubating the slides in 10mM citrate buffer at 90°C for 20min and then washed 2×3 min in PBS. Primary antibody incubation was carried out overnight at 4°C with one of the following primary antibodies in 2% normal donkey serum and 0.1% Triton X-100 in PBS: rat anti-mouse CD11b (AbD Serotec (MCA711), 1:1000); rabbit anti-mouse TSPO (Abcam (EPR5384), 1:250); rabbit anti-mouse GFAP (DAKO (Z0334), 1:1000); mouse anti-human 6E10 amyloid (BioLegend (803001) 1:1000); rabbit anti-mouse SV2A (Abcam (ab32942) 1:500); chicken anti-mouse MAP2 (Abcam (ab5392), 1:1000); rabbit anti-mouse NeuN (Abcam (ab177487), 1:500); rabbit anti-mouse Neurogranin (Abcam (ab23570), 1:500). Following incubation in primary antibody, PBS washes were repeated (3×10min) and incubated with one of the following secondary antibodies was carried out: Alexa Fluor 594nm Donkey anti-rat IgG 1:500 (for CD11b); Alexa Fluor 488nm Donkey anti-rabbit IgG 1:500 (for TSPO, GFAP, SV2A); Alexa Fluor 594nm Donkey anti-mouse IgG 1:500 (for 6E10); Alexa Fluor 488nm Goat anti-chicken Double staining was carried out for CD11b+TSPO, CD11b+GFAP and 6E10+TSPO and to allow the visualisation of microglia and astrocytes with TSPO expression and Aβ burden. Double staining was also carried out for MAP2+NeuN to look at neuronal density.
